# Linking Hematopoietic Differentiation to Co-Expressed Sets of Pluripotency-Associated and Imprinted Genes and to Regulatory microRNA-Transcription Factor Motifs

**DOI:** 10.1371/journal.pone.0166852

**Published:** 2017-01-04

**Authors:** Mohamed Hamed, Johannes Trumm, Christian Spaniol, Riccha Sethi, Mohammad R. Irhimeh, Georg Fuellen, Martina Paulsen, Volkhard Helms

**Affiliations:** 1 Center for Bioinformatics, Saarland University, Saarbrücken, Germany; 2 Institute for Biostatistics and Informatics in Medicine and Ageing Research, Rostock University Medical Center, Rostock, Germany; 3 Kolling Institute of Medical Research, Royal North Shore Hospital, The University of Sydney, St Leonards, NSW, Australia; 4 Department of Genetics, Saarland University, Saarbrücken, Germany; National Institute of Technology Rourkela, INDIA

## Abstract

Maintenance of cell pluripotency, differentiation, and reprogramming are regulated by complex gene regulatory networks (GRNs) including monoallelically-expressed imprinted genes. Besides transcriptional control, epigenetic modifications and microRNAs contribute to cellular differentiation. As a model system for studying the capacity of cells to preserve their pluripotency state and the onset of differentiation and subsequent specialization, murine hematopoiesis was used and compared to embryonic stem cells (ESCs) as a control. Using published microarray data, the expression profiles of two sets of genes, pluripotent and imprinted, were compared to a third set of known hematopoietic genes. We found that more than half of the pluripotent and imprinted genes are clearly upregulated in ESCs but subsequently repressed during hematopoiesis. The remaining genes were either upregulated in hematopoietic progenitors or in differentiated blood cells. The three gene sets each consist of three similarly behaving gene groups with similar expression profiles in various lineages of the hematopoietic system as well as in ESCs. To explain this co-regulation behavior, we explored the transcriptional and post-transcriptional mechanisms of pluripotent and imprinted genes and their regulator/target miRNAs in six different hematopoietic lineages. Therewith, lineage-specific transcription factor (TF)-miRNA regulatory networks were generated and their topologies and functional impacts during hematopoiesis were analyzed. This led to the identification of TF-miRNA co-regulatory motifs, for which we validated the contribution to the cellular development of the corresponding lineage in terms of statistical significance and relevance to biological evidence. This analysis also identified key miRNAs and TFs/genes that might play important roles in the derived lineage networks. These molecular associations suggest new aspects of the cellular regulation of the onset of cellular differentiation and during hematopoiesis involving, on one hand, pluripotent genes that were previously not discussed in the context of hematopoiesis and, on the other hand, involve genes that are related to genomic imprinting. These are new links between hematopoiesis and cellular differentiation and the important field of epigenetic modifications.

## Introduction

The maintenance of cellular pluripotency, the onset of differentiation as well as cellular differentiation into particular lineages appear to be controlled by tightly regulated gene regulatory networks (GRNs) that describe the interactions between transcription factors (TFs) and microRNAs and their target genes [1]. For example, Fuellen and co-workers have manually compiled from the original literature a dataset of murine genes termed the PluriNetwork that are involved in the regulation of the pluripotent state [2]. Besides transcriptional control, epigenetic modifications such as DNA methylation and histone marks are increasingly gaining attention with respect to cellular differentiation. One of the hallmarks of epigenetics is the phenomenon of genomic imprinting, which describes parent-of-origin monoallelic expression [3]. As the importance of epigenetic modes of gene regulation is particularly evident for imprinted genes, these genes serve as common model systems. Therefore, we are focusing here on the expression patterns and modes of regulation of the genes that have been shown to be monoallelically expressed in the mouse.

Hematopoiesis describes the differentiation of hematopoietic stem cells (HSCs) into lineage-committed progenitor cells. Recent transcriptomics studies have identified important parts of the regulatory networks that control maintenance of HSCs [4] and progenitors [1, 5, 6]. During hematopoiesis, little is known about the imprinting status of imprinted genes. As an exception to this, maternal imprinting at the H19-Igf2 locus was shown to maintain adult hematopoietic stem cell quiescence [7]. Additionally, several lines of evidence exist for the importance of imprinted genes during the transition from the stem cell stage to differential commitment as well as during particular cell lineages, namely hematopoiesis. For example, a network of 15 co-regulated imprinted genes involved in embryonic growth has been identified [8]. Ten of these genes were downregulated in terminally differentiated mouse cells compared to long-term repopulating HSCs [9]. In multipotent progenitor cells, 13 out of 15 imprinted genes were clearly downregulated compared to HSC whereas the two imprinted genes *Gnas* and *Gatm* were upregulated in MPP3 and MPP4 relative to MPP1 and HSC [5]. Recently, we identified 10 imprinted genes that are transcriptionally regulated by the hematopoiesis-related TF NFAT. We also found 9 imprinted genes that are targets of the *FOXO4* TF [10]. In CD34^+^ cells, the imprinted maternally expressed gene *p57* (*Cdkn1c)* was the only cyclin-dependent kinase inhibitor to be rapidly upregulated by TGFβ, a negative regulator of hematopoiesis [11]. Additionally, we found that promoter regions around the transcription start sites of *Mkrn3*, *Igf2*, and *Gnas* genes contain DNA motifs that match to annotated binding site motifs for the TFs *E2A* and *TCF3 [10]*. The latter plays major roles in determining tissue-specific cell fate during embryogenesis such as early B-cell lymphopoiesis and germinal center B-cell development [12]. Several studies from the Li group indicated that the expression of certain imprinted genes changes in HSCs during differentiation from quiescent to multi-lineage progenitors [13]. However, the transcriptional and post-transcriptional activities as well as the interwoven regulatory mechanisms of imprinted genes and imprinting-related miRNAs (that are regulators/targets of imprinted genes) in the onset and further progression of cell differentiation (for the example of hematopoiesis) and the aspects of their involvement have not been addressed in detail before.

Identifying regulatory elements that are related to the differentiation of hematopoietic cells from the pluripotent stage into a lineage committed stage is of great importance to the fields of stem cell biology and hematology. The role of imprinted and pluripotent genes in hematopoiesis as compared to embryonic stem cells (ESC) and their expression patterns was not previously analyzed or studied in a systemic manner. Therefore, we conducted this study to investigate the functional connections of sets of genes in hematopoiesis and differentiation. We constructed lineage-specific regulatory networks involving imprinted genes, pluripotent genes, related miRNAs, and central hubs that could act as lineage drivers. Our results were validated via various analytical approaches and their biological relevance was demonstrated in explaining the differentiation process of HSCs that is tightly regulated through a set of imprinted, pluripotent and hematopoiesis-specific genes, providing a better understanding of the regulatory mechanisms of cellular differentiation of blood cells.

## Materials and Methods

### Gene selection

Three mouse gene lists were prepared (imprinted, pluripotent, and hematopoietic genes). Our selection of imprinted genes was not done manually as in some of our previous work [10, 14, 15] as the manually curated lists contained a rather restricted number of genes. In this study, our selection was based on the overlap of several well-known online catalogs of imprinted genes. Imprinted genes were downloaded from four well-known catalogs [IGC database (http://www.otago.ac.nz/IGC) [16], Geneimprint (http://www.geneimprint.com/site/genes-by-species.Musmusculus), WAMIDEX (https://atlas.genetics.kcl.ac.uk) [17], and MouseBook^TM^ (http://www.mousebook.org/catalog.php?catalog=imprinting)]. A single list of 120 genes (called henceforth candidate imprinted genes) was created from the four catalogs by including only genes that appeared in at least two catalogs and by filtering out genes that have conflicting or unknown imprinting status in the various catalogs (*i*.*e* whether they are maternally or paternally expressed) (Table A in S1 File). As this is a computational study, we did not verify experimentally whether these genes are actually monoallelically expressed in the investigated cell lines or not.

The pluripotent gene list including 274 genes was obtained from the PluriNetWork [2], a hand curated pluripotency-controlling gene network in mouse with 574 regulatory interactions. To the best of our knowledge, no generally accepted GRN for the global hematopoiesis system has been established. In the absence of such a model, the 615 genes that are annotated in the Gene Ontology [18] for the GO term *hematopoietic or lymphoid organ development* (GO:0048534) were considered as ‘hematopoiesis genes’. Not all genes in the three gene lists were annotated in the Affymetrix array. Of the 120 imprinted genes only 86 were annotated (the rest were mostly non-coding RNAs, which are thus not considered), whereas only 2 out of 274 pluripotent genes and 53 out of the 615 hematopoietic genes were not annotated. The counts of overlapping genes are shown in Figure A in S1 File.

### Microarray analysis

Gene expression microarray data of three hematopoietic datasets (accession IDs GSE6506 [19], GSE14833 [20], GSE34723 [21]) and one non-hematopoietic dataset (control) (GSE10246 [22]) that also contains ESC samples, all based on the Affymetrix GeneChip Mouse Genome 430 2.0 Array, were downloaded from the Gene Expression Omnibus (http://www.ncbi.nlm.nih.gov/geo/) [23]. Data normalization, model-based expression measurements, and annotation of the imprinted and pluripotent genes to their corresponding probes in the four datasets were done using the GC-RMA method and mouse 4302.db packages, respectively, by using the Bio-conductor software [24] within the statistical programming language R [25].

A gene expression similarity score was calculated to measure how similar the normalized expression of an individual gene present on the chip is to the distribution of normalized expression values for the sets of pluripotent and hematopoietic genes, separately across the four datasets. Let us consider, for example, the similarity to pluripotent genes. In that case, the binned expression value of a gene *g*_*i*_ in each cell sample *s*_*j*_ was weighted by the number *PD*_*sj*_ of pluripotent genes having expression values in the same bin in sample *s*_*j*_. To reduce statistical noise, the GC-RMA normalized expression data was fitted by Gaussian curves. The respective values were then used as *PD*_*sj*_ values.

This product was summed over all samples to give a representative similarity score for each gene. A detailed illustration is shown in the supplementary Figure B in S1 File.

$$\begin{array}{c} SimScore\;(g_{i})=\sum_{j=1}^{cell\;samples}{PD}_{sj}\times g_{i,sj}\;with\;i\in [1,\;number\;of\;imprinted\;genes]\\ and\;j\in [1,\;all\;cell\;samples\;per\;dataset] \end{array}$$

Next, we separated the similarity scores of imprinted genes and non-imprinted genes and examined with the Mann-Whitney U-test whether imprinted genes have a higher gene expression similarity to pluripotent and hematopoietic genes than the background of all other genes. Additionally, we defined top scored genes as the highest 10% of the ranked genes and then applied the hyper-geometric test to investigate the significance of having imprinted genes among the defined top scored genes. We also tested the null distribution hypothesis by randomly shuffling the expression values of the imprinted genes and recalculating the similarity scores. The procedure was repeated 1000 times and the p-value was computed based on the number of random times in which the similarity scores were higher than the real score without shuffling.

For lineage specificity, six isometric lineages (three lymphoid and three myeloid) were constructed from the four expression datasets by following the hematopoietic differentiation model in Seita *et al*., [21], (Table 1). We looked at three main hematopoiesis developmental stages: early progenitors (LTHSC and STHSC), intermediate progenitors (LMPP and CLP), and terminally differentiated blood cells (MKE and GM). Then we used a conservative differential expression approach based on moderated t-test to encode the differences between the three stages. P-values were adjusted using the Benjamini-Hochberg procedure [26] to limit the false discovery rate to 5%. Lineages that are constructed from dataset GSE6506 and contain only two stages (early progenitors and terminally differentiated cells) were analyzed by setting an on/off expression threshold (similar to Chambers *et al*., [19]) to identify uniquely expressed genes in each stage of the cell development of each lineage. Finally, a gene was confirmed as differentially expressed gene if it appeared in the same lineage in at least two different datasets.

**Table 1:** Selected hematopoietic lineages and their stages of sequential cell development.

Lineage	Sequential Cell Development
**B-cell**	*HSC→ MPPa → MPPb→ GMLPa → GMLPb → CLP→ BLP→ PREPROB → FrB→ FrC → FrD → FrE*
**NK-cell**	*HSC→ MPPa→ MPPb→ GMLPa→ GMLPb→ CLP→ iNK→ mNK*
**T-cell**	*HSC→ MPPa→ MPPb→ GMLPa→ GMLPb→ CLP→ DN1→ DN2→ DN3a→ DN3b→ DN4→ DPCD69*^*-*^ *→ DPCD69*^*+*^*→ CD4*^*+*^*CD69*^*+*^ *→ CD4*^*+*^*CD69*^*-*^
**Erythrocytes**	*HSC→ MPPa → pMEP → MEP → pCFU*–*E*
**Monocytes**	*HSC → MPPa → sCMP→ pGMPa→ pGMPb → GMP → Mono*
**Granulocytes**	*HSC→ MPPa → sCMP → pGMPa → pGMPb → GMP → Gra*

*HSC*, Hematopoietic Stem Cells; *MPPa*, Multipotent Progenitor state A; *MPPb*, Multipotent Progenitor state B; *GMLPa*, Granulocyte Macrophage Lymphoid Progenitor state A; *GMLPb*, Granulocyte-Macrophage-Lymphoid Progenitor state B; *CLP*, Common Lymphoid Progenitor; BLP, Earliest B Lymphoid Progenitor; *PREPROB*, Precursor of B-cells Progenitor; FrB, Fraction B B-cell; FrC, Fraction C B-cell; FrD, Fraction D B-cell; FrE, Fraction E B-cell; iNK, intermediate Natural Killer Cell; mNK, mature Natural Killer Cell; DN1, Double Negative T-cell 1; DN2, Double Negative T-cell 2; DN3a, Double Negative T-cell 3a; DN3b, Double Negative T-cell 3b; DN4, Double Negative T-cell 4; *DPCD69*^*-*^, Double Positive CD69^-^ T-cell; *DPCD69*^*+*^, Double Positive CD69^+^ T-cell*; CD4*^*+*^*CD69*^*+*^, CD4^+^ CD69^+^ T-cell; *CD4*^*+*^*CD69*^-^, CD4^+^ CD69^-^ T-cell; *MEP*, Megakaryocyte/ Erythrocyte Progenitor; *pMEP*, pre of *MEP*; *pCFU*-*E*, pre of *CFU*-*E*; *sCMP*, Strict Common Myeloid Progenitor; *GMP*, Granulocyte-Macrophage-Progenitor; *pGMPa*, pre of GMP state A; *pGMPb*, pre of GMP state B; *Mono*, Monocytes; Gra, Granulocytes.

### miRNA Enrichment

We predicted the miRNAs associated with the list of differentially expressed mRNAs of each lineage by determining the set of miRNAs whose target genes as well as regulator TFs are significantly enriched within each differentially expressed gene set. For this, we used the hypergeometric distribution function followed by the Benjamini-Hochberg adjustment with a cutoff value of 0.001. The TF *→* miRNA interactions and miRNA *→* target gene interactions were compiled from the regulatory databases listed in [27].

### Construction of TF-miRNA regulatory networks and motif modules

The integrated association between the lineage markers and the enriched miRNAs were compiled from the regulatory databases of the murine TFmiR webserver [27]. All molecular interactions that are supported either by experimental and/or by predicted evidences were considered in this analysis. Next, 3-node FFL motifs (TF-miRNA co-regulatory networks consisting of a miRNA, a TF, and a joint target gene) were characterized using the computational procedure described in [27]. To identify critical network players, we computed the degree centrality measure (number of links incident upon a node) for each lineage network using the R package igraph [28] and highlighted the top 10% highest centrality nodes of the TFs/genes and miRNAs. The networks and co-regulatory motifs were visualized with Cytoscape V3.3.0 [29].

### Network validation and assessment of key player nodes

#### 1- Statistical validation: significance of the detected motifs

To evaluate the significance of each co-regulatory motif, we compared how often it appears in the lineage regulatory network against the number of times it appears in randomized versions of these networks with preserved node degrees. The randomization procedure is explained in detail in [27]. The random networks were constructed *N*_*r*_ = 100 times and compared to the real network. The p-value was calculated as

$$p–value=\frac{N_{h}}{N_{r}}$$

where *N*_*h*_ is the number of times that a certain motif type is identified in a randomized network at equal or higher number than in the lineage network. Only motifs having p-value < 0.05 were considered for further analysis.

#### 2- Semantic validation: functional homogeneity within the motif nodes

In order to assess the biological relevance of the identified TF–miRNA co-regulatory motifs and to better understand their functional roles, we analyzed the GO semantic similarity for all pairs of target genes regulated either by the TF or by the miRNA of each lineage-specific TF–miRNA motif. The GoSemSim R package [30] was used to calculate the semantic similarity scores accordingto the Gene Ontology (GO) annotations. Statistical significance was determined by randomly selecting the same number of co-regulated genes (genes targeted by either the TFs or the miRNA) from all Entrez genes with GO annotations, and computing their similarity scores. The permutation procedure was repeated 100 times. Then, we utilized the Kolmogorov-Smirnov test to check whether the functional similarity scores of all gene pairs composing a regulatory motif are significantly higher than that of randomly selected pairs.

#### 3- Enrichment Analysis for genes and miRNAs

The functional annotation tool in DAVID [31] was used to identify significantly enriched functional categories in the gene sets. For this, we determined which GO categories were annotated to at least 2 genes and are statistically overrepresented in the co-expressed genes against the full mouse genome (control) as previously shown in [10]. Then, enrichment was evaluated through the Fisher Exact test using p-value threshold of 0.05. Enrichment analysis of the miRNAs sets was performed using the TAM online tool [32] as previously done in [33].

#### 4- Literature support

We used the PubMed database to search for evidence-based hematopoietic lineage commitment and cellular differentiation. Network nodes (TFs/genes and miRNAs) with such evidence supported by at least one published article are highlighted in the network visualization.

## Results and Discussion

In this study, we re-analyzed published gene expression microarray data deposited in GEO [23] [three hematopoietic datasets (accession IDs GSE6506 [19], GSE14833 [20], GSE34723 [21]) and one data set that is not specific to hematopoiesis (control) (GSE10246 [22])]. This was done in order to identify regulatory elements and their transcriptional and post-transcriptional interactions that are related to the transition of cells from the pluripotent stem cell stage into the lineage-committed stage. As explained in the methods section, we established three lists of imprinted, pluripotent, and hematopoietic genes. It is important to note that some genes in the pluripotent list might be directly involved in maintaining the pluripotency of embryonic stem (ES) cells and induced pluripotent stem cells (iPS), whereas some other genes might have indirect and more general functionalities, such as cell cycle regulation etc. From these lists, 86 imprinted, 272 pluripotent, and 562 hematopoietic genes are annotated on the microarray.

We start with a compact overview of the different stages of our computational analysis. First, the expression patterns of the imprinted and pluripotent genes during different stages of the hematopoietic lineages were compared to the global expression patterns of hematopoietic genes. We identified marker genes that are exclusively differentially expressed in each of the six main hematopoietic lineages and we postulated their functional roles by scrutinizing the mammalian phenotypes associated with hematopoietic abnormalities. Then, we predicted for each lineage marker gene set the miRNAs with significantly enriched target genes and significantly enriched regulatory TFs of the marker genes. Using regulatory data sources, we constructed lineage-specific ‘TF-miRNA-mediated regulatory networks’. In these networks, we identified 3-node Feed Forward Loop (FFL) motifs consisting of imprinted/pluripotent TFs and co-targeted genes as well as imprinting/pluripotency-associated miRNAs. Finally, we validated/tested the contribution of these TF-miRNA co-regulatory motifs to the transcriptional activities of the corresponding hematopoietic lineage in terms of statistical significance and their association with biological evidence. It turned out that many miRNAs and TFs/genes of the identified lineage-specific co-regulatory motifs were implicated by previous work in the sequential cell development of either the related hematopoietic lineage or hematopoiesis in general. Our results demonstrate that the constructed lineage-specific mediated regulatory networks (comprising imprinting- and pluripotency-associated TFs and miRNAs) contain valuable new information for researchers that may aid in identifying critical TFs, miRNAs, and their targets for further experimental design, and enhance the understanding of the regulatory mechanisms of hematopoietic cellular differentiation.

### Imprinted genes show similar expression patterns to pluripotent and hematopoietic genes

**Fig 1:**
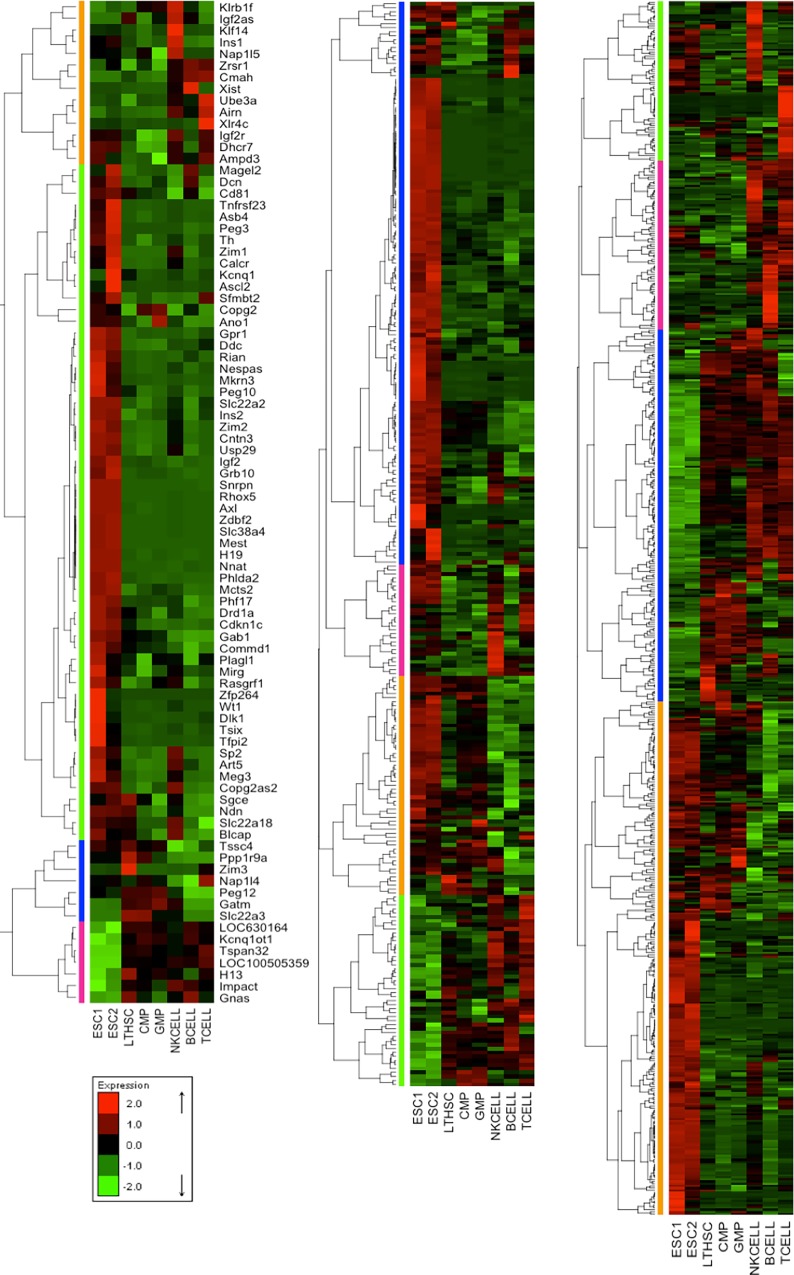
Heatmaps showing transient changes in expression profiles. Different groups of ESC and hematopoietic cells (*e*.*g* stem cells, intermediate progenitors, and terminally differentiated blood cells) from the GSE10246 dataset for (left panel) imprinted genes, (middle panel) pluripotent genes and (right panel) hematopoietic genes were compared. Green spots represent downregulated genes, and red spots represent upregulated genes. The order of genes is obtained by hierarchical clustering, which shows three similar pattern classes between imprinted, pluripotent and hematopoietic genes.

To provide an overview, Fig 1 shows clustered normalized expression profiles for two ESC lines, three progenitor cell lines (Long Term HSC: LTHSC, Common Myeloid Progenitor: CMP, and Granulocyte-Macrophage-Progenitor: GMP), and three terminally differentiated cell lines (NK-cells, B-cells, T-cells). Clustering as well as visual inspection revealed three main classes of expression patterns, which are shared by most imprinted, pluripotent and hematopoietic genes. The first class (1^st^) contains genes that have high expression levels in ESC and have gradually decreasing expression levels during the two stages of hematopoiesis (early and intermediate progenitors and terminally differentiated blood cells). More than half of the imprinted genes (Fig 1, left panel, green) and of the pluripotent genes (Fig 1, middle panel, blue) belong to this class (1^st^). Also, about one third of the hematopoiesis genes (Fig 1, right panel, orange) belong to the same 1^st^ class. Genes of the second class (2^nd^) are characterized by overexpression in the early and intermediate progenitors (more specifically in Common Lymphoid Progenitor: *CLP*) and have relatively low expression levels in both ESC and terminally differentiated cells. The third class (3^rd^) includes genes that are predominantly upregulated in matured blood cells. Interestingly, the 2^nd^ and 3^rd^ classes contain genes from all three gene sets. On the basis of gene ontology (GO) annotation, we investigated the functional similarity of the three genes sets among each other and with respect to randomly selected genes (Figure C in S1 File). This analysis revealed that pluripotent genes and hematopoietic genes share the highest similarity of GO annotation (about 0.75). This is quite expected since the genes from both sets are involved in regulating cell fate. Comparing the functional similarity of the pluripotent genes of the 1^st^ class to the hematopoiesis genes of the 1^st^ class resulted in slightly higher numbers than measuring the functional similarity between mixed classes. In comparison, the average functional similarity of imprinted genes with pluripotent genes or with hematopoiesis genes was lower (about 0.6), but still clearly higher than that with randomly selected genes (about 0.3).

To quantify this visual impression, we ranked all genes according to their gene expression similarity score (see methods section) across all considered hematopoietic samples. This score measures how similar the expression of an individual gene is to the distribution of expression values for the sets of pluripotent and hematopoietic genes. Notably, all p-values for the three hematopoietic datasets (that encompass differentiation and cell development data only) were significant (between 0.001 and 0.01) (Table 2). Moreover, a large portion of imprinted genes was found to belong to the highest 10% of the ranked genes in GSE6506 and GSE34723 datasets (66% and 59%, respectively, Table 2). In contrast, no significant difference was found between the ranking of imprinted genes and the background of all genes of the control (GSE10246; largely non-hematopoietic) and the number of top ranked imprinted genes was lowest here. Many imprinted genes shared very similar expression patterns with the pluripotent and hematopoietic gene sets (Fig 1, Table 2) similar to observations made for a smaller set of imprinted genes in murine HSCs [9]. This similarity was most pronounced in 1^st^ class genes that are overexpressed in ESCs. However, the functional similarity of imprinted genes, pluripotent, and hematopoietic genes does not quite reach the level that is seen for the similarity between PluriNetWork and hematopoiesis genes. We attribute the observation that particular imprinted genes show a high variability of expression among the various stages of differentiation to the different roles played by these genes in the cell.

**Table 2:** Statistical comparison of gene expression similarity (gene similarity scores).

Dataset	Compared genes to background	Mann-Whitney U-Test	Permutation Test	Top Scored Imprinted Genes	Hyper-geometric Test
**GSE6506** [19]	**Pluripotent**	0.006	0.019	55	0.006
	**Hematopoietic**	0.044	0.032	57	0.010
**GSE34723** [21]	**Pluripotent**	0.004	0.022	50	0.004
	**Hematopoietic**	0.003	0.006	51	0.009
**GSE14833** [20]	**Pluripotent**	0.003	0.014	18	0.195 *
	**Hematopoietic**	0.006	0.072 *	24	0.214 *
**GSE10246** [22] **(Control)**	**Pluripotent**	0.106	0.267	11	0.784
	**Hematopoietic**	0.101	0.089	14	0.700

First, the Mann-Whitney U-test was used to test whether imprinted genes have a higher gene expression similarity to pluripotent and hematopoietic genes than to the background of all other genes (non-imprinted genes). Then the top 10% scoring genes were tested using hyper-geometric test to find out the significance of having imprinted genes among them (rightmost column). Secondly, also a permutation test was performed to test the null distribution hypothesis by randomly shuffling the expression values of the imprinted genes and recalculating the similarity scores. The procedure was repeated 1000 times. The p-value was computed based on the number of random times where the similarity scores were higher than the real score without shuffling. (*) Among the three hematopoietic datasets, only the p-value of the hyper-geometric test for GSE14833 and the respective permutation test does not meet the significance threshold of 0.05.

**Fig 2:**
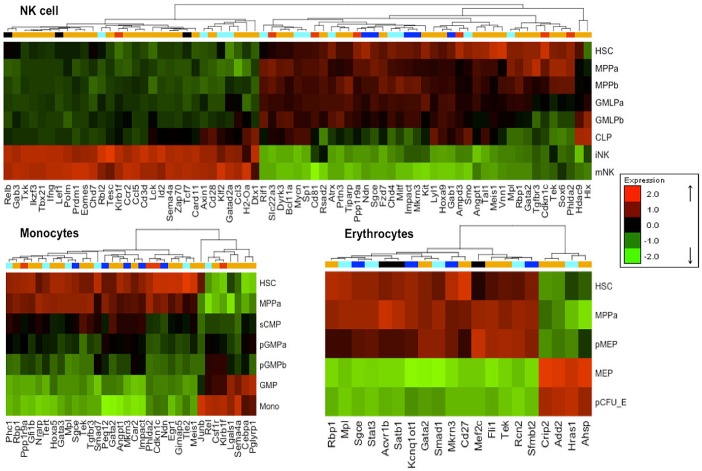
Heatmaps of differentially expressed imprinted genes. The order of genes is obtained by hierarchical clustering of three blood lineages (NK-cells, Monocytes, and Erythrocytes) based on the GSE34723 dataset. Gene clustering color coding is (blue) for paternally expressed genes, (red) for maternally expressed, (cyan) for pluripotent genes, and (orange) for hematopoietic genes. Shared genes between the pluripotent and hematopoietic gene sets are marked in black. Green spots represent downregulated genes, and red spots represent upregulated genes. The clustering reveals that for every lineage, there exist imprinted as well pluripotent and hematopoietic genes showing similar expression changes during cell development. The other three lineages (B-cells, T-cells, and granulocytes) are shown in the supplementary Figure D in S1 File.

Comparing the expression levels of known pluripotent with hematopoietic genes showed that the compiled PluriNetWork [2] contains not only the GRN that keeps cells in the pluripotent state but appears also to be related to the regulation of the onset of cellular differentiation such as hematopoiesis. In fact, the GO terms *hematopoietic or lymphoid organ development*, *haemopoiesis*, *myeloid cell differentiation*, *leukocyte differentiation* are annotated to 44, 39, 27, and 23 pluripotent genes, respectively, suggesting that a significant portion of the pluripotent genes is indeed involved in hematopoiesis regulation (Table C in S1 File). Moreover, Figs 1 and 2 demonstrate convincingly that the full set of pluripotent genes displayed pronounced variations during different stages of hematopoiesis and in individual cell lineages as well. These findings agree with previous studies that discussed the role of pluripotent genes in determining cell fate and controlling differentiation [34].

### All three gene sets contribute to hematopoietic lineage specificity

With a focus on the differentiation along particular lineages, we subjected the selected microarray datasets to differential expression analysis. For this, we divided cell samples into three classes [early progenitors (*e*.*g HSC* and *MPP*), intermediate progenitors (*e*.*g GMLP* and *CLP)*, and terminally differentiated blood cells (*e*.*g Monocytes* and *NK-cells*)] so that this analysis was now based on far more cell types than the global analysis in Fig 1. Table B in S1 File lists those imprinted, pluripotent, and hematopoietic genes having statistically significantly different expression patterns during the sequential stages of specific blood cell lineages.

Lineage-specific differentially expressed genes (here termed marker genes) found in the three gene sets and the related expression heatmaps for NK-cells, monocytes, and erythrocytes are shown in Fig 2 (heatmaps for the other 3 lineages are shown in Figure D in S1 File). The number of significant lineage markers varies between 23 genes (in granulocytes) and 193 genes (in B-cells). Only the three genes *Rbp1*, *Sgce* and *Mkrn3* (the last two are imprinted genes) are shared by all myeloid branches (erythrocytes, monocytes, and granulocytes) (Table B in S1 File). A recent study found that the expression of *Rbp1* was affected significantly by the medical indication of acute myeloid leukemia (AML) [35]. Additionally, we identified 16 marker genes (*e*.*g Lgals1*, *Gimap5*, *Pml*, and *Hoxa5*) that are exclusively differentially expressed in myeloid lineages (not in lymphoid). These 16 genes are annotated for terms such as GO:0002317 "*plasma cell differentiation*", GO:0043011" *myeloid dendritic cell differentiation*”, GO:0030099 “*myeloid cell differentiation*”, and GO:0045639 “*positive regulation of myeloid cell differentiation*”, respectively. Along the same lines, the lymphoid markers contain 30 genes shared by all lymphoid peers (B-cell, T-cell, and NK-cell) and 226 genes that were only detected for individual lymphoid lineages (not myeloid) such as *Tcf7*, *Lef1*, and *Rel*. The latter gene plays a role in differentiation and lymphopoiesis [36]. A complete list of the identified genes is given in Table B in S1 File.

Different cell types showed pronounced differences in their gene expression profiles: most prominent was the high number of differentially expressed genes in B-cells and T-cells with a major contribution of pluripotent genes and to some extent also imprinted genes. This expression profile is interesting as a substantial portion of B-cells and T-cells serve as memory cells that can be induced by secondary infections to undergo further cell divisions [37, 38]. NK-cells that have recently been shown to have some potential for further cell divisions [39] tend to have more differentially expressed genes compared to the differentiated myeloids. Many of the lineage markers identified in this work are concordant with the findings of previous work. Generally, the ten lineage markers (*Cdkn1c*, *Ndn*, *Gatm*, *Phlda2*, *Air*, *Igf2r*, *Slc22a3*, *H13*, *Sfmbt2*, and *Peg12*) that participate in most lineages were demonstrated to be differentially expressed in the early onset of the hematopoietic process [7, 40]. More specifically, the identified erythrocytes lineage markers *Fli1*, *Mpl*, and *Gata2* were previously found to determine the erythrocytes signature [5, 41–43].

Separate labeling of maternally and paternally expressed genes did not reveal a clear-cut separation, which is consistent with previous findings [10]. Nevertheless, only paternally expressed genes were differentially expressed in the erythrocyte lineage (Fig 2). In contrast, the imprinted genes that were overexpressed during late stages of hematopoiesis tend to be maternally expressed (*e*.*g Cmah* and *Nap1l4* in B- and T-cells, *Klrb1f* in monocytes and NK-cells, *Th* and *Igf2r* in T-cells) rather than paternally expressed (*Sp2*, *Mcts2*, and *Ddc* only in B-cells and T-cells). Three imprinted genes (*Ndn*, *Peg3*, *and Peg12*) that were annotated by Chambers and colleagues [19] as HSC specific genes were identified here as marker genes for differentiated lineages. Consistent with the findings of Chambers *et al*., they are highly expressed in HSCs and downregulated in differentiated states.

The postulated functional role of the identified lineage markers during hematopoiesis was backed up by inspecting the mammalian phenotypes associated with hematopoiesis abnormalities using the MGI database [44], (Table B in S1 File). Apparently, deleting the lineage-specific marker genes leads to deficiencies in either functionalities or differentiation of a specific lineage, validating the used approach in identifying the lineage markers. An example from the B-cell lineage is the knockout of the imprinted gene *CD81*. This is reported to cause *abnormal B-cell morphology* (MGI ID: MP:0004939), *decreased B-1 B-cell number* (MP:0004978), and *instability in B-cell proliferation* (MP:0005154, MP:0005093). More generally, the knockout of the imprinted gene *Cdkn1c* leads to *decreasing hematopoietic stem cell number* (MP:0004810) and *abnormal hematopoietic stem cell physiology* (MP:0010763). From the set of pluripotent genes, gene knockout of *Relb* exhibits also several abnormalities such as *decreased B-cell number* (MP:0005017), *decreased B-cell proliferation* (MP:0005093), *absent lymph nodes* (MP:0008024), *decreased pre-B-cell number* (MP:0008209), and *extra-medullary hematopoiesis* (MP:0000240).

### Construction of TF-miRNA mediated regulatory network and hub genes

Subsequently, we identified the miRNAs associated with each marker gene set and constructed for each lineage a GRN network that represents the combinatorial regulatory mechanisms of miRNAs and TFs (see Material and Methods) to the extent these are reflected in the regulatory databases that this work was based on. Each such lineage network contains three types of nodes, namely miRNAs, TFs, and target genes. In order to assess the contribution of each node type in the lineage–specific GRN networks to their stability and robustness, we computed the node degree centrality parameters and ranked the nodes according to their degrees.

The most connected nodes (miRNAs and TFs and target genes) are candidates for central hubs (key network players) that could possibly drive the cellular switch towards the related hematopoietic lineage commitment and thus could act as potential master regulator. In Table 3, we list the top 10% nodes with highest degree for each lineage. For instance, we identified 5 hub TFs/genes (*Rel*, *Ppp1r9a*, *Smad7*, *Ndn*, *and Cdkn1c*) and 10 hub miRNAs (*mir-92a-3p*, *mir-377-3p*, *mir-17-5p*, *mir-96-5p*, *mir-875-3p*, *mir-875-5p*, *mir-721*, *mir-330-3p*, *mir-20a-5p*, *and mir-19a-3p*) for the monocyte lineage (Figure E in S1 File). Among them, genes *Rel*, *Smad7*, *and Cdkn1c* had been reported to be essential TFs in cell fate determination of hematopoietic progenitors of monocytes [45–48] and the absence of the *Rel* gene leads to multiple hematopoietic cell defects and apoptosis during monocyte differentiation [45]. Moreover, we assessed the biological role of the identified hub nodes by linking them to functional and disease annotations via overrepresentation analysis (ORA). We identified the significant functional terms and associated diseases that were enriched in the group of identified hub miRNAs of each lineage network, see Table 3. Strikingly, in 5 out of 6 lineages, the identified hub miRNAs were enriched with hematopoiesis-related biological processes such as *angiogenesis* (p-value < 0.0017), *embryonic stem cell regulation* (p-value < 0.00071), *cell fate determination* (p-value < 0.017002), *immune system* (p-value < 2.47e-7) as well as with hematopoietic lineage-specific diseases such as leukemia T-Cell (p-value < 4.1e-7), and Lymphoma B-Cell (p-value < 1.06e-4). The networks constructed for monocytes and granulocytes and their hub genes/miRNAs are visualized in Figures E and F in S1 File, respectively.

**Table 3:** The identified central hub nodes (TFs/miRNAs) and their enrichment analysis.

Lineage	Hub TFs/genes	Related enriched Go Terms	Hub miRNAs	Related enriched Functional terms and diseases (p-value)
**B-cell**	Pou2f1, Zfx, Creb1, Myc, Klf4, Mycn, Rel, Rela, Dnmt3a, Satb2, Ppp1r9a, Mtf2, Phf17, Ccnd1, Rbbp7, Pbrm1, Smad2, Sgk1, Smarca4, Acvr1, Smad4, Mitf, Dnmt1, Smarca2, Hdac2, Terf2, Il6st, Smarcad1, Ctbp2, Smarcc1, Mapk1, H3f3a, Mta2, Ewsr1, Fgf4, Ndn, Dhx9, Rcn2, Hdac1, Gab1, Acvr1b, Irs1, Smad1, Lef1, Chd4, Zfp143, Tcf3, Klf2, Arid3b, Bmpr2, Ehmt2, Mkrn3, Fgfr1, Sfmbt2, Gatm, Sumo1, Cdkn1c, Relb, Sp2, Cd81, Stk40, Carm1, Cdk2, Hira, Axl, Ssrp1, Smarca5, Parp1	•Positive regulation of B-cell proliferation (0.009) •Embryonic pattern specification (0.01) •Cell fate commitment (0.0046) •Cell differentiation (1,6e-7) •Embryonic morphogenesis (6.7e-8) •Cell-cell signaling involved in cell fate specification (0.011)	mmu-mir-19a-3p, mmu-mir-19b-3p, mmu-mir-92a-3p, mmu-mir-495-3p, mmu-mir-17-5p, mmu-mir-200c-3p, mmu-mir-9-5p, mmu-mir-302b-3p, mmu-mir-302a-3p, mmu-mir-20a-5p, mmu-mir-381-3p, mmu-mir-302d-3p, mmu-mir-137-3p, mmu-mir-124-3p, mmu-mir-497-5p, mmu-mir-32-5p, mmu-mir-200b-3p, mmu-mir-195a-5p, mmu-let-7i-5p, mmu-mir-875-3p, mmu-mir-694, mmu-mir-340-5p, mmu-mir-338-5p, mmu-mir-30e-5p, mmu-mir-30b-5p	•Embryonic stem cell regulation (0.0047) •Cell fate determination (0.00039) •Cell proliferation (Hwang et al BJC 2006) (5.2e-7) •Hematopoiesis (0.0071) •Onco-miRNA (0.007) Leukemia, B-Cell (0.000034) •Leukemia, Myeloid (0.000004) •Lymphoma, B-Cell (0.000106) •Lymphoma, Large-Cell, Anaplastic (0.00000456)
**T-cell**	Ctcf, Creb1, Mycn, Trp53, Foxd3, Atf2, Mef2c, Ppp1r9a, Ccnd1, Il6st, Hif1a, Mitf, Terf2, Smad2, Kdm6b, Igf2r, Ndn, Socs1, Ctbp2, Gab1, Kdm6a, Acvr1b, Gatm, Smad1, Ewsr1, Rcn2, Arid3b, Sfmbt2, Smurf1, Dhx9, Smad3, Mta2, Mkrn3, Cdkn1c, Bmpr2, Stk40, Sp2, Klf2, Lef1, Ehmt2, H13, Spp1, Notch1, Cdk2, Ocln, Satb1, Zfp219, Tcf7, Pim1, Hdac1, Cd81, Fgfr1, Hira, Grb2, Impact, Tcf3, Med12, Axin1	•Fat cell differentiation (0.023) •Embryonic pattern specification (0.008) •Immune system development (0.005) •Leukocyte differentiation (0.017) •hemopoiesis (0.016) •B-cell lineage commitment (0.019) •hemopoietic or lymphoid organ development (0.023)	mmu-mir-381-3p, mmu-mir-19b-3p, mmu-mir-19a-3p, mmu-mir-20a-5p, mmu-mir-17-5p, mmu-mir-30e-5p, mmu-mir-302d-3p, mmu-mir-302b-3p, mmu-mir-30c-5p, mmu-mir-302a-3p, mmu-mir-291b-3p, mmu-mir-20b-5p, mmu-mir-124-3p, mmu-mir-106b-5p, mmu-mir-106a-5p, mmu-mir-96-5p, mmu-mir-93-5p, mmu-mir-92a-3p, mmu-mir-32-5p, mmu-mir-30b-5p, mmu-mir-30a-5p, mmu-mir-291a-3p, mmu-mir-497-5p	•Angiogenesis (0.001768) •Embryonic stem cell regulation (0.00071201) •cell fate determination (0.017002) •Hematopoiesis (0.005842) •Immune system(Xiao's et al Cell 2009) (2.47e-7) •Adipocyte differentiation (0.003) •Hematologic Neoplasms (0.00000138) •Leukemia-Lymphoma, Adult T-Cell (4.1e-7) •Lymphoma, T-Cell (0.00000138)
**NK-cell**	Mycn, Sp1, Ppp1r9a, Atrx, Ndn, Rbl2, Mitf, Gab1, Klf2, Cd81, Chd4, Cdkn1c, Tcf7, Mkrn3, Impact, Relb, Gatad2a, Lef1	•hemopoiesis (0.035) •myeloid leukocyte differentiation (0.039) •immune system development (0.047) •myeloid cell differentiation (0.0057)	mmu-mir-92a-3p, mmu-mir-381-3p, mmu-mir-25-3p, mmu-mir-20a-5p, mmu-mir-200c-3p, mmu-mir-17-5p, mmu-mir-92b-3p, mmu-mir-367-3p, mmu-mir-363-3p, mmu-mir-32-5p, mmu-mir-200b-3p	•Akt pathway (0.0036) •Angiogenesis (0.01) •Hematopoiesis (0.02) •Immune system (0.000009) •Hormones regulation (0.004) •Cell proliferation (0.0001) •Leukemia, Myeloid (0.0003) •Lymphoma, Large-Cell, Anaplastic (0.00033) •Hematologic Neoplasms (0.0002)
**Monocyte**	Rel, Ppp1r9a, Smad7, Ndn, Cdkn1c	•cell development (0.01) developmental process (0.0245) •neuron differentiation (0.004) •regulation of metabolic process (0.029)	mmu-mir-92a-3p, mmu-mir-377-3p, mmu-mir-17-5p, mmu-mir-96-5p, mmu-mir-875-5p, mmu-mir-875-3p, mmu-mir-721, mmu-mir-330-3p, mmu-mir-20a-5p, mmu-mir-19a-3p	•Granulopoiesis (0.013) •Cell proliferation (2.9e-7) •Hematopoiesis (0.00052) •Angiogenesis (0.000186) •Leukemia, Myeloid, Acute (0.00131697) •Myeloproliferative Disorders (0.0018) •Hematologic Neoplasms (6.8e-7)
**Granulocyte**	Ppp1r9a, Ndn, Mkrn3	•Cell projection organization (0.044) •Neuron development (0.04)	—	—
**Erythrocyte**	Stat3, Mef2c, Rcn2, Smad1, Sfmbt2, Acvr1b, Mkrn3, Satb1, Hras1	-Cell differentiation (0.033) -Positive regulation of macromolecule biosynthetic process (0.0257) -Cellular macromolecule metabolic process (0.037) -Organ morphogenesis (0.031)	mmu-mir-26b-5p, mmu-mir-495-3p, mmu-mir-26a-5p, mmu-mir-93-5p, mmu-mir-467e-3p, mmu-mir-466a-3p, mmu-mir-329-3p, mmu-mir-291b-5p, mmu-mir-291a-5p, mmu-mir-223-3p, mmu-mir-219-5p, mmu-mir-199a-3p, mmu-mir-183-5p, mmu-mir-135a-5p, mmu-mir-106b-5p, mmu-let-7c-5p, mmu-let-7b-5p, mmu-mir-96-5p, mmu-mir-92b-3p, mmu-mir-9-5p, mmu-mir-880-3p, mmu-mir-878-3p, mmu-mir-762	•Embryonic stem cell (hESC) regulation (0.009) •Cell division (0.013) •Cell fate determination (0.00005) •immune system (0.015) •miRNA tumor suppressor (0.02) •Anti-cell proliferation (0.043) •Leukemia, Myeloid, Acute (0.027) •melanoma (0.00807) •Lymphoma, Primary Effusion (0.043)

### Identification of TF-miRNA co-regulatory motifs

**Fig 3:**
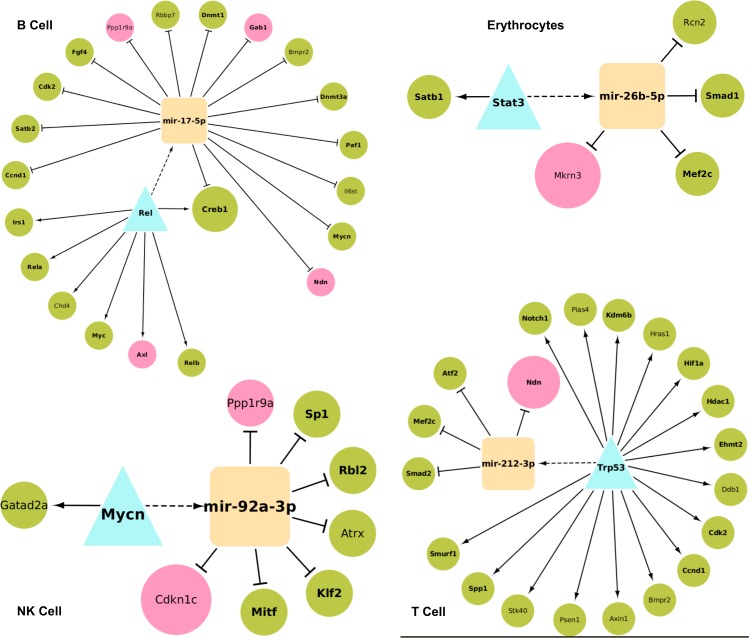
The most significant TF-miRNA co-regulatory motifs for 4 hematopoietic lineages. TFs are represented by a turquoise triangle whereas miRNAs are shown as orange squares. Green circles denote the pluripotent genes whereas imprinted genes are colored in pink. Bold label nodes are supported by literature evidence.

Transcriptional regulation networks often contain Feed Forward Loop (FFL) motifs as functional interconnection patterns [49] that govern many aspects of normal cell functions and diseases [50, 51]. Here, we checked for the presence of 3-node co-regulatory FFL motifs involving the lineage marker TFs/genes and miRNAs in the GRNs of individual hematopoietic lineages (see Materials and Methods). We found that such 3-node motifs were significantly enriched only in the B-cell, T-cell, erythrocyte, and NK-cell lineage networks. The statistical significance of the motifs was tested by comparing their count in the real network to their counts in randomized variants of these networks preserving the same node degrees. In the B-cell, T-cell, erythrocyte, and NK-cell lineages 1727, 1182, 11, and 60 statistically significant FFL motifs were detected, respectively. Fig 3 illustrates the most significant motif detected in each lineage network. Interestingly, these motifs involved both imprinted and pluripotent genes as either a TF or a target gene suggesting the collaborative role and their participation in cellular differentiation and development in the corresponding lineages. A good example for that is the detected B-cell motif that comprises the miRNA mir-17-5p, the imprinted genes *Axl*, *Ndn*, *Gab1*, *Ppp1r9a* and the pluripotent genes *Rel*, *Ccnd1*, *Satb2*, *Creb1*, *Cdk2*, *Fgf4*, *Rbbp7*, *Dnmt1*, *Bmpr2*, *Dnmt3a*, *Paf1*, *Il6st*, *Mycn*, *Myc*, *Chd4*, *Rela*, *Irs1*, *Relb*. Noteworthy is the TF *Rel* gene that was characterized as a hub gene of the B-cell lineage (Table 3) and was reported to play a critical role in cell fate determination of multiple hematopoietic progenitors including B-cell [45]. Also, the TF *Myc* belongs to the four known Yamanaka factors that play a significant role in cell reprogramming [52] and were shown to be sufficient for reprogramming differentiated cells into iPS [53]. *Myc* is believed to regulate the expression of 15% of all human genes [54] and plays an important role in B-cell proliferation [55]. The imprinted gene *Gab1* was found to recruit cytosolic signaling proteins to cellular membranes where they can act on membrane-bound targets during the activation of multiple B-cell antigen receptor signaling pathways [56].

Another example is the erythrocytes motif that involves the pluripotent TF *Stat3* (which was previously reported to be essential in the regulation of erythropoiesis and erythrocytes development [57]) and the miRNA mir-26b-5p (which exhibits moderate changes during erythroid differentiation [58]). The TF regulates the miRNA which in turns regulates the imprinted gene *Mkrn3* whose expression level was significantly increased in several hematopoietic lineages with distinct patterns [59].

**Fig 4:**
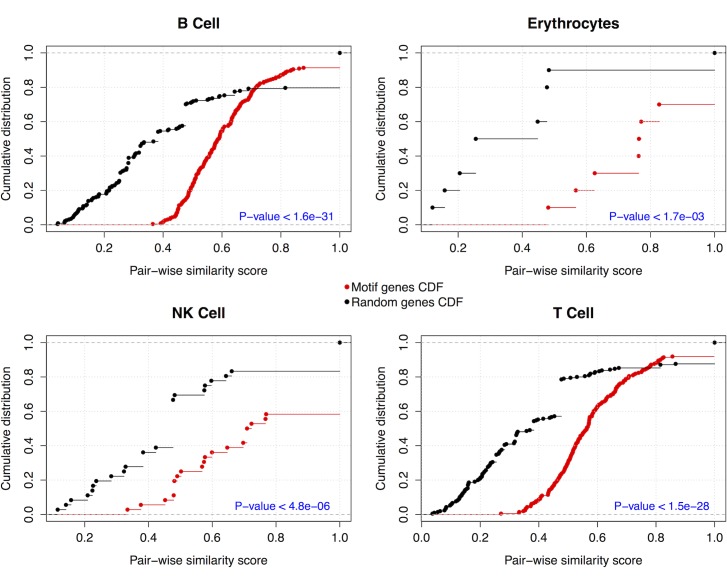
Functional homogeneity of the identified co-regulatory motifs. Cumulative distribution of GO functional semantic scores of gene pairs of co-regulated genes in the examined motifs (red) versus randomly selected genes (black). The p-value was calculated using the Kolmogorov-Smirnov test.

Furthermore, we evaluated the biological evidence of these motifs to better understand their functional roles in the development of lineage cell states. For this, we computed the functional similarity scores between co-regulated gene pairs as a measure of their functional homogeneity and integrity. The distribution of the resulting similarity scores was compared to the similarity score distribution of randomly selected gene pairs (p-values < 0.00173, Kolmogorov-Smirnov test), see Fig 4. As the co-regulated genes have significantly more similar cellular functions than randomly selected genes, these FFL motifs could provide novel insights into TF-miRNA networks during lineage commitment and specification and suggest a cooperative functional role between the TFs/genes and potential miRNA partners. Especially when combined with experimental validation, these co-regulatory motifs as well as the identified hub TFs/genes and miRNAs could expand our understanding of prospective lineage-specific genomic drivers and therefore could help to better understand the cell fate determination of hematopoietic progenitor cells.

In summary, we unraveled specific molecular regulatory interactions involving imprinted genes that explain how these genes contribute to cellular differentiation processes and to different developmental stages of hematopoiesis. The presented analysis suggests new aspects of the cellular regulation of the onset of cellular differentiation and during hematopoiesis. These involve, on the one hand, pluripotent genes that were previously not discussed in the context of hematopoiesis and, on the other hand, involve imprinted genes that are related to genomic imprinting.

## Supporting Information

S1 File**Figure A. Venn diagram of the 3 gene sets involved in the analysis**. Imprinted, pluripotent, and hematopoietic genes. **Figure B. A sketch diagram showing the calculation of the similarity score between the two observation groups:** 1- the similarity scores between imprinted genes and (pluripotent or hematopoietic) genes and 2- the similarity scores between non-imprinted genes and (pluripotent or hematopoietic) genes as a background. The expression values of the imp/non-imp genes are weighted by the Gaussian fitting of the expression values of the corresponding pluripotent/ hematopoietic sample. **Figure C. The functional similarity between genes belonging to the first class (c1) of each gene set in Fig 1 in comparison to 2 background sets.** In bg1, class 1 of the first gene set is compared with "not class 1" of the other gene set. In bg2, class 1 of the first gene set is compared with a randomly selected set of genes from the other gene set that has the same size like class 1. Therefore the following labels are used in the plot:

**Table:** 

imp_hema:	"imp(c1) vs hema(c1)",
i_h(bg1):	"imp(c1) vs hema (notc1)",
i_h(bg2):	"imp(c1) vs random genes of size hema c1"
imp_pluri:	"imp(c1) vs pluri(c1)",
i_p(bg1):	"imp(c1) vs pluri (notc1)",
i_p(bg2):	"imp(c1) vs random genes of size pluri c1",
plur_hema:	"pluri(c1) vs hema(c1)",
p_h(bg1):	"pluri(c1) vs hema (notc1)",
p_h(bg2):	"pluri(c1) vs random genes of size hema c1"**Figure D. Heatmaps of differentially expressed imprinted genes (paternally expressed are in blue and maternally expressed are in red), pluripotency genes (cyan), and hematopoietic genes (orange) along three blood lineages (B cells, T cells, and granulocytes) based on GSE34723 dataset.** The other three lineages are shown in Fig 2. Shared genes between pluripotency and hematopoietic gene sets are marked in black. Green spots represent down-regulated genes, and red spots represent up-regulated genes. The clustering reveals that for every developmental line, there exist imprinted as well pluripotency and hematopoietic genes showing similar expression changes during development. **Figure E. A TF-miRNA network for the monocyte lineage.** Green color denotes the pluripotent genes whereas imprinted genes are colored in pink. miRNAs are colored in orange. Darker nodes are the identified putative driver TFs/miRNAs. Bold label nodes are nodes, which are supported by literature evidence. **Figure F. A TF-miRNA network for the granulocyte lineage.** Green color denotes the pluripotent genes whereas imprinted genes are colored in pink. miRNAs are colored in orange. Darker nodes are the identified putative driver TFs/miRNAs. Bold label nodes are nodes, which are supported by literature evidence. **Table A in S1 File. List of the 86 imprinted genes that are annotated in the MS4302.0 microarray chip. Table B in S1 File. List of lineage-specific imprinted, pluripotent, and hematopoietic genes.** in the investigated blood lineages and the associated mammalian phenotypes due to gene knock outs according to the MGI database. **Table C in S1 File. Enrichment of Pluripotency genes with Hematopoietic functional terms.**(DOCX)
