# Supplementary material for: Linking Hematopoietic Differentiation to Co-Expressed Sets of Pluripotency-Associated and Imprinted Genes and to Regulatory microRNA-Transcription Factor Motifs
Source: PLoS One. 2017 Jan 4;12(1):e0166852. doi: 10.1371/journal.pone.0166852 (PMC5215400; doi:10.1371/journal.pone.0166852)
Supplement: S1 File — Figure A. Venn diagram of the 3 gene sets involved in the analysis. Imprinted, pluripotent, and hematopoietic genes. Figure B. A sketch diagram showing the calculation of the similarity score between the two observation groups: 1- the similarity scores between imprinted genes and (pluripotent or hematopoietic) genes and 2- the similarity scores between non-imprinted genes and (pluripotent or hematopoietic) genes as a background. The expression values of the imp/non-imp genes are weighted by the Gaussian fitting of the expression values of the corresponding pluripotent/ hematopoietic sample. Figure C. The functional similarity between genes belonging to the first class (c1) of each gene set in Fig 1 in comparison to 2 background sets. In bg1, class 1 of the first gene set is compared with "not class 1" of the other gene set. In bg2, class 1 of the first gene set is compared with a randomly selected set of genes from the other gene set that has the same size like class 1. Therefore the following labels are used in the plot: 10.1371/journal.pone.0166852.t004imp_hema:"imp(c1) vs hema(c1)",i_h(bg1):"imp(c1) vs hema (notc1)",i_h(bg2):"imp(c1) vs random genes of size hema c1"imp_pluri:"imp(c1) vs pluri(c1)",i_p(bg1):"imp(c1) vs pluri (notc1)",i_p(bg2):"imp(c1) vs random genes of size pluri c1",plur_hema:"pluri(c1) vs hema(c1)",p_h(bg1):"pluri(c1) vs hema (notc1)",p_h(bg2):"pluri(c1) vs random genes of size hema c1" Figure D. Heatmaps of differentially expressed imprinted genes (paternally expressed are in blue and maternally expressed are in red), pluripotency genes (cyan), and hematopoietic genes (orange) along three blood lineages (B cells, T cells, and granulocytes) based on GSE34723 dataset. The other three lineages are shown in Fig 2. Shared genes between pluripotency and hematopoietic gene sets are marked in black. Green spots represent down-regulated genes, and red spots represent up-regulated genes. The clustering reveals that for every developmental line, there ex [file pone.0166852.s001.docx]

**Supplementary Files**

**Fig A.**

**
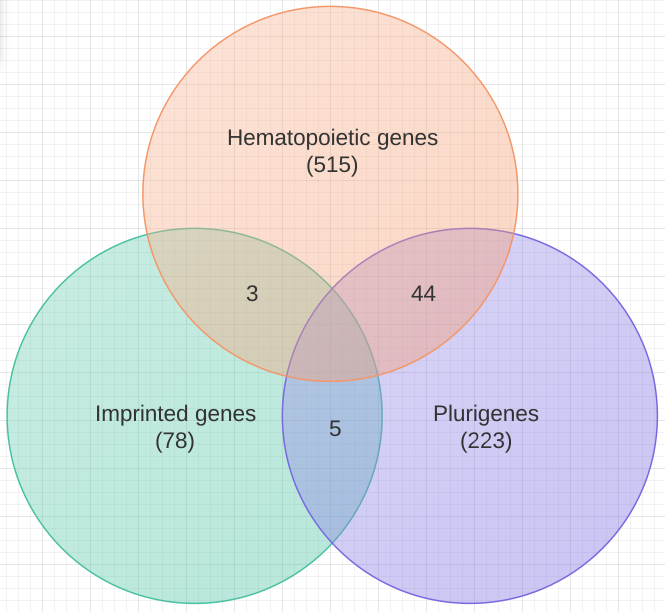
**

**Fig B.**

**Fig C.**

**
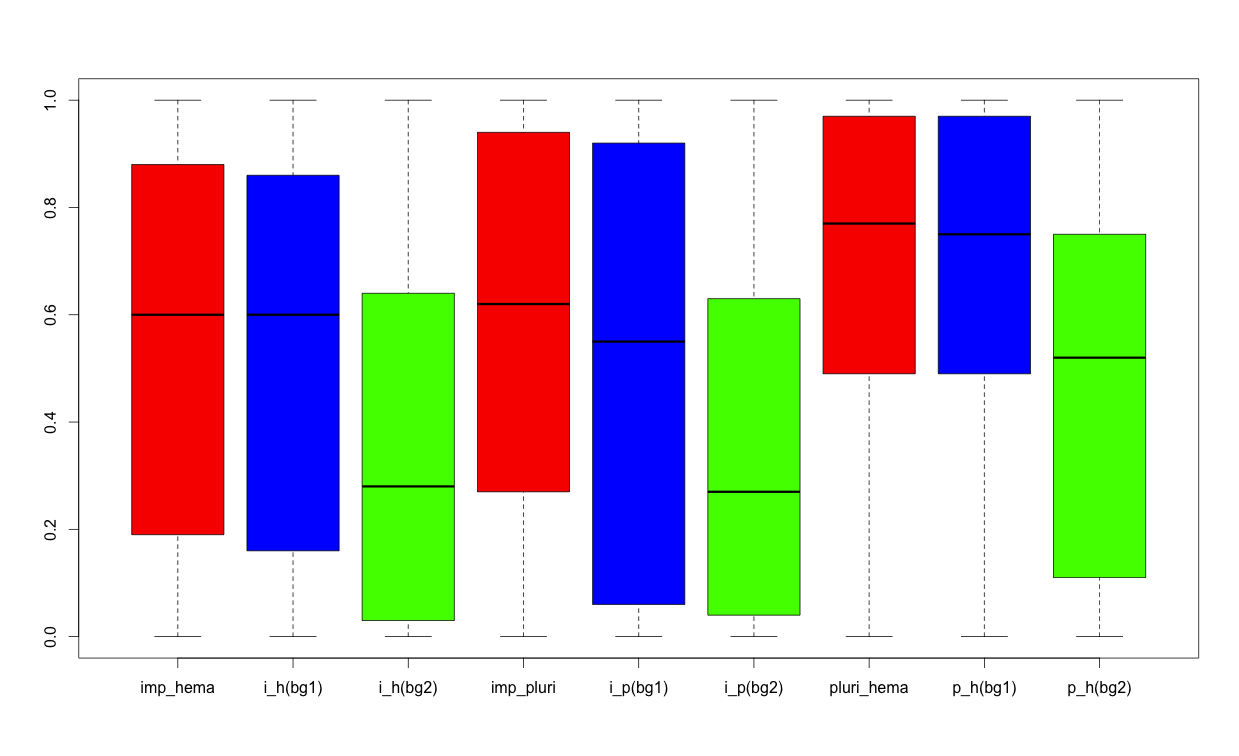
**

**Fig D.**

**
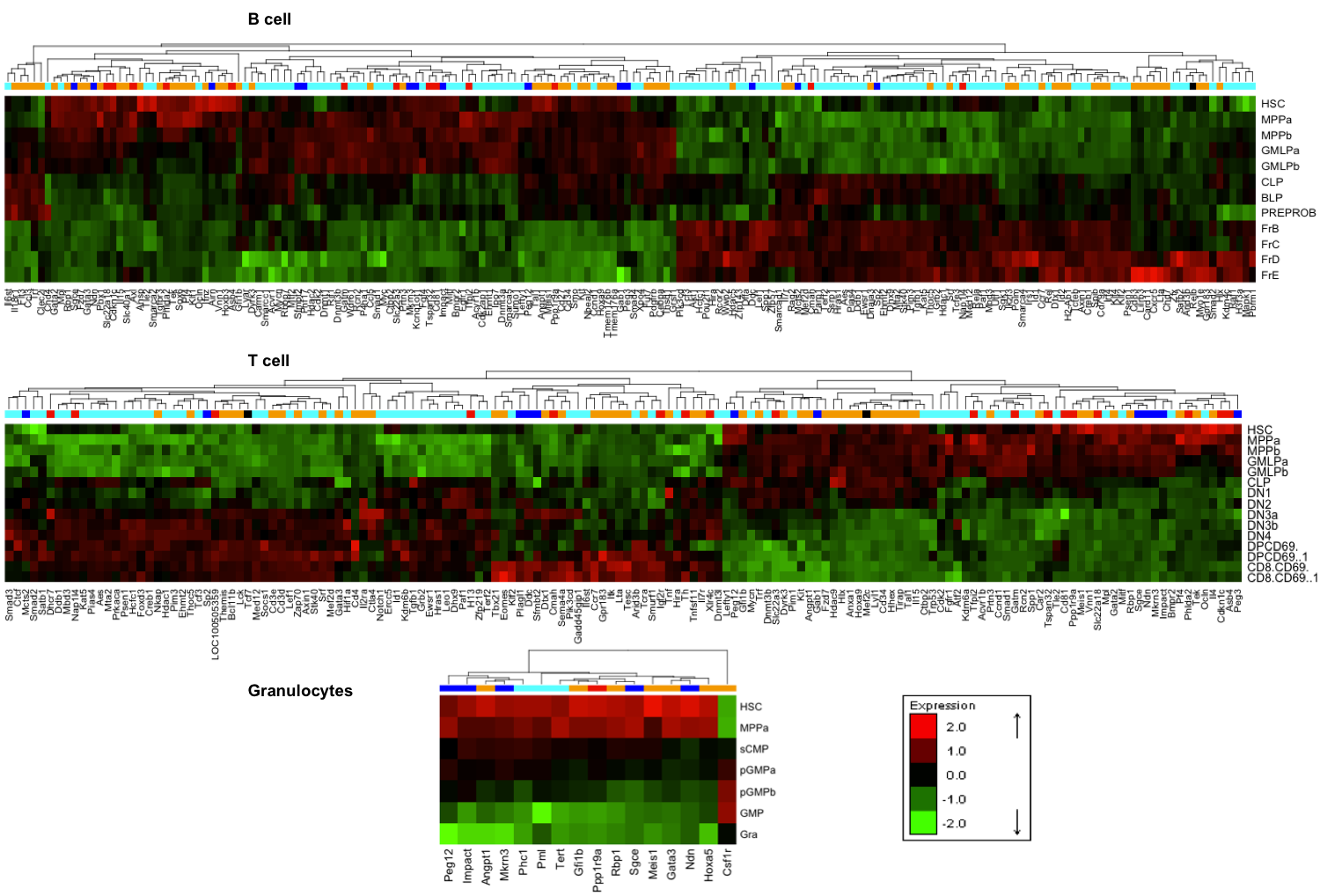
**

**Fig E.**

**
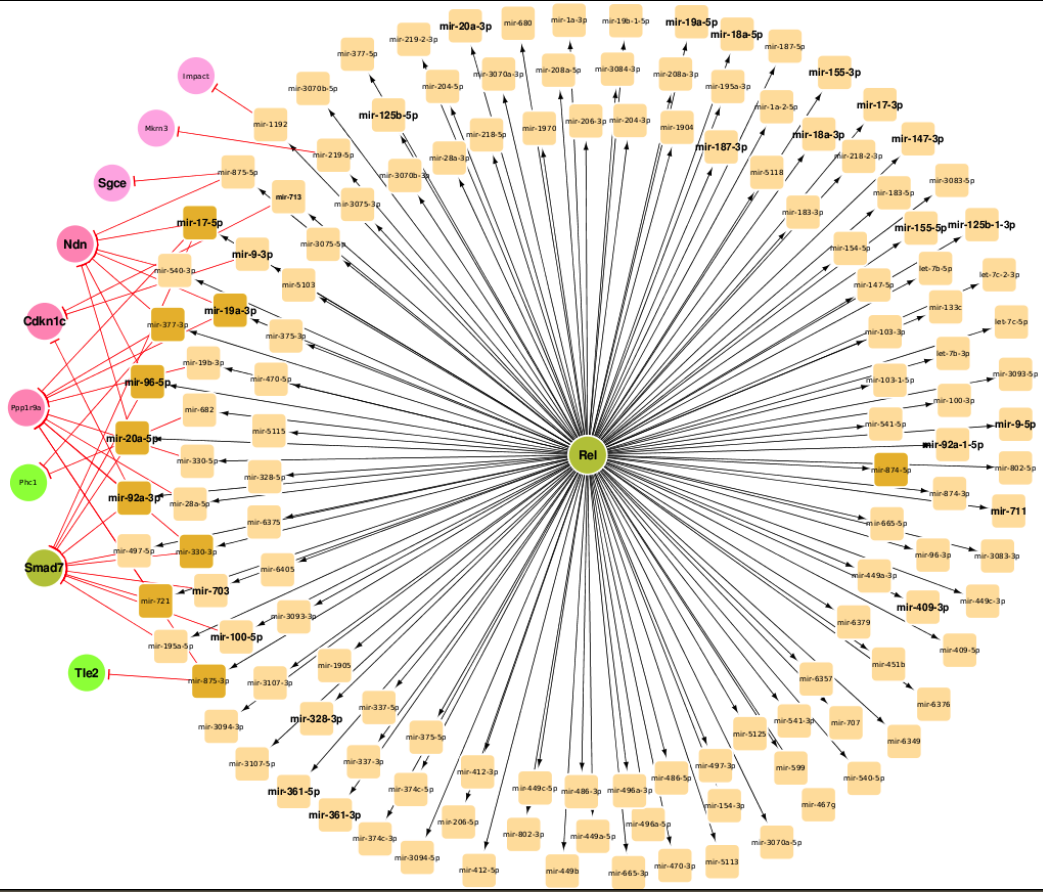
**

**Fig F.**

**Table A.**

| **Gene symbol** | **Gene Title** | **Entrez ID** | **RefSeq Transcript ID** |
| --- | --- | --- | --- |
| Airn /// Air | antisense Igf2r RNA | 104103 | NR_002853 /// NR_027772 /// NR_027773 /// NR_027784 |
| Ampd3 | adenosine monophosphate deaminase 3 | 11717 | NM_009667 |
| Ano1 | anoctamin 1, calcium activated chloride channel | 101772 | NM_178642 |
| Art5 | ADP-ribosyltransferase 5 | 11875 | NM_007491 |
| Asb4 | ankyrin repeat and SOCS box-containing 4 | 65255 | NM_023048 |
| Ascl2 | achaete-scute complex homolog 2 (Drosophila) | 17173 | NM_008554 |
| Axl | AXL receptor tyrosine kinase | 26362 | NM_001190974 /// NM_001190975 /// NM_009465 |
| Blcap | bladder cancer associated protein homolog (human) | 53619 | NM_016916 |
| Calcr | calcitonin receptor | 12311 | NM_001042725 /// NM_007588 |
| Cd81 | CD81 antigen | 12520 | NM_133655 |
| Cdkn1c | cyclin-dependent kinase inhibitor 1C (P57) | 12577 | NM_001161624 /// NM_009876 |
| Cmah | cytidine monophospho-N-acetylneuraminic acid hydroxylase | 12763 | NM_001111110 /// NM_007717 |
| Cntn3 | contactin 3 | 18488 | NM_008779 |
| Commd1 | COMM domain containing 1 | 17846 | NM_144514 |
| Copg2 | coatomer protein complex, subunit gamma 2 | 54160 | NM_017478 |
| Copg2as2 | coatomer protein complex, subunit gamma 2, antisense 2 | 100044236 | NR_002845 |
| Dcn | decorin | 13179 | NM_001190451 /// NM_007833 |
| Ddc | dopa decarboxylase | 13195 | NM_001190448 /// NM_016672 |
| Dhcr7 | 7-dehydrocholesterol reductase | 13360 | NM_007856 |
| Dlk1 | delta-like 1 homolog (Drosophila) | 13386 | NM_001190703 /// NM_001190704 /// NM_001190705 /// NM_010052 /// NR_033813 |
| Drd1a | dopamine receptor D1A | 13488 | NM_010076 |
| Gab1 | growth factor receptor bound protein 2-associated protein 1 | 14388 | NM_021356 |
| Gatm | glycine amidinotransferase (L-arginine:glycine amidinotransferase) | 67092 | NM_025961 |
| Gnas /// Nesp | GNAS (guanine nucleotide binding protein, alpha stimulating) complex locus | 14683 | NM_001077507 /// NM_001077510 /// NM_010309 /// NM_019690 /// NM_022000 /// NM_201616 /// NM_201617 /// NM_201618 /// NR_003258 |
| Gpr1 | G protein-coupled receptor 1 | 241070 | NM_146250 |
| Grb10 /// Meg1 | growth factor receptor bound protein 10 | 14783 | NM_001177629 /// NM_010345 |
| H13 | histocompatibility 13 | 14950 | NM_001159551 /// NM_001159552 /// NM_001159553 /// NM_010376 |
| H19 | H19 fetal liver mRNA | 14955 | NR_001592 |
| Igf2 | insulin-like growth factor 2 | 16002 | NM_001122736 /// NM_001122737 /// NM_010514 |
| Igf2as | insulin-like growth factor 2, antisense | 111975 | NR_002855 |
| Igf2r | insulin-like growth factor 2 receptor | 16004 | NM_010515 |
| Impact | imprinted and ancient | 16210 | NM_008378 |
| Ins1 | insulin I | 16333 | NM_008386 |
| Ins2 | insulin II | 16334 | NM_001185083 /// NM_001185084 /// NM_008387 |
| Kcnq1 | potassium voltage-gated channel, subfamily Q, member 1 | 16535 | NM_008434 |
| Kcnq1ot1 | KCNQ1 overlapping transcript 1 | 63830 | NR_001461 |
| Klf14 | Kruppel-like factor 14 | 619665 | NM_001135093 |
| Klrb1f | killer cell lectin-like receptor subfamily B member 1F | 232408 | NM_153094 /// NR_024262 /// NR_024263 |
| LOC100505359 /// Xlr4a /// Xlr4b /// Xlr4c | x-linked lymphocyte-regulated protein 3A-like /// X-linked lymphocyte-regulated 4A /// X-linked lymphocyte-regulated 4B /// X-linked lymphocyte-regulated 4C | 100505359 /// 27083 /// 434794 /// 72891 | NM_001081642 /// NM_021365 /// NM_183094 /// XM_003085319 /// XM_003085320 /// XM_003085321 /// XM_003085322 |
| LOC630164 /// Xlr3a /// Xlr3b /// Xlr3c | x-linked lymphocyte-regulated protein 3B-like /// X-linked lymphocyte-regulated 3A /// X-linked lymphocyte-regulated 3B /// X-linked lymphocyte-regulated 3C | 22445 /// 22446 /// 574437 /// 630164 | NM_001081643 /// NM_001110784 /// NM_011727 /// XM_903265 |
| Magel2 | melanoma antigen, family L, 2 | 27385 | NM_013779 |
| Mcts2 | malignant T cell amplified sequence 2 | 66405 | NM_025543 |
| Meg3 | maternally expressed 3 | 17263 | NR_003633 /// NR_027651 /// NR_027652 |
| Mest /// Peg1 | mesoderm specific transcript | 17294 | NM_008590 |
| Mirg | MiRNA containing gene | 100040724 | NR_028265 |
| Mkrn3 | makorin, ring finger protein, 3 | 22652 | NM_011746 |
| Nap1l4 | nucleosome assembly protein 1-like 4 | 17955 | NM_008672 |
| Nap1l5 | nucleosome assembly protein 1-like 5 | 58243 | NM_021432 |
| Ndn | necdin | 17984 | NM_010882 |
| Nespas | neuroendocrine secretory protein antisense | 56802 | NR_002846 |
| Nnat | neuronatin | 18111 | NM_010923 /// NM_180960 |
| Peg10 | paternally expressed 10 | 170676 | NM_001040611 /// NM_130877 |
| Peg12 | paternally expressed 12 | 27412 | NM_013788 |
| Peg3 | paternally expressed 3 | 18616 | NM_008817 |
| Phf17 | PHD finger protein 17 | 269424 | NM_001130184 /// NM_001130185 /// NM_001130186 /// NM_172303 |
| Phlda2 | pleckstrin homology-like domain, family A, member 2 | 22113 | NM_009434 |
| Plagl1 /// Zac1 | pleiomorphic adenoma gene-like 1 | 22634 | NM_009538 |
| Ppp1r9a | protein phosphatase 1, regulatory (inhibitor) subunit 9A | 243725 | NM_181595 |
| Rasgrf1 | RAS protein-specific guanine nucleotide-releasing factor 1 | 19417 | NM_001039655 /// NM_011245 |
| Rhox5 | reproductive homeobox 5 | 18617 | NM_008818 |
| Rian | RNA imprinted and accumulated in nucleus | 75745 | NR_028261 |
| Sfmbt2 | Scm-like with four mbt domains 2 | 353282 | NM_001198808 /// NM_001198809 /// NM_177386 |
| Sgce | sarcoglycan, epsilon | 20392 | NM_001130188 /// NM_001130189 /// NM_001130190 /// NM_001130191 /// NM_011360 |
| Slc22a18 | solute carrier family 22 (organic cation transporter), member 18 | 18400 | NM_001042760 /// NM_008767 |
| Slc22a2 | solute carrier family 22 (organic cation transporter), member 2 | 20518 | NM_013667 |
| Slc22a3 | solute carrier family 22 (organic cation transporter), member 3 | 20519 | NM_011395 |
| Slc38a4 | solute carrier family 38, member 4 | 69354 | NM_027052 |
| Snrpn /// Snurf | small nuclear ribonucleoprotein N /// SNRPN upstream reading frame | 20646 /// 84704 | NM_001082961 /// NM_001082962 /// NM_013670 /// NM_033174 |
| Sp2 | Sp2 transcription factor | 78912 | NM_001080964 /// NM_030220 |
| Tfpi2 | tissue factor pathway inhibitor 2 | 21789 | NM_009364 |
| Th | tyrosine hydroxylase | 21823 | NM_009377 |
| Tnfrsf23 | tumor necrosis factor receptor superfamily, member 23 | 79201 | NM_024290 |
| Tsix | X (inactive)-specific transcript, antisense | 22097 | NR_002844 |
| Tspan32 | tetraspanin 32 | 27027 | NM_001128080 /// NM_001128081 /// NM_001128082 /// NM_020286 |
| Tssc4 | tumor-suppressing subchromosomal transferable fragment 4 | 56844 | NM_001115085 /// NM_020285 /// NM_138631 |
| Ube3a | ubiquitin protein ligase E3A | 22215 | NM_001033962 /// NM_011668 /// NM_173010 |
| Usp29 | ubiquitin specific peptidase 29 | 57775 | NM_021323 |
| Wt1 | Wilms tumor 1 homolog | 22431 | NM_144783 |
| Xist | inactive X specific transcripts | 213742 | NR_001463 /// NR_001570 |
| Xlr4c | X-linked lymphocyte-regulated 4C | 72891 | NM_183094 |
| Zdbf2 | zinc finger, DBF-type containing 2 | 73884 | NM_028673 |
| Zfp264 | zinc finger protein 264 | 116812 | XM_912760 |
| Zim1 | zinc finger, imprinted 1 | 22776 | NM_011769 |
| Zim2 | zinc finger, imprinted 2 | 76637 | XM_489216 /// XM_922461 |
| Zim3 | zinc finger, imprinted 3 | 116811 | NR_036631 |
| Zrsr1 | zinc finger (CCCH type), RNA binding motif and serine/arginine rich 1 | 22183 | NM_011663 |
| **Non annotated imprinted genes in the microarray chip:** Zfp127as, Hymai, Rtl1, Dio3, Htr2a, Kcnk9, Peg13, Inpp5f_v2, Copg2as1, Apeg3, Pec2, Pec3, Pihit, Fbxo40, Gtl2, Mkrn1-ps1, Mir335, Af313042, Mir184, Mir380, Mir370, Mir376b, Mir410, Mir154, Mir134, Mir127, Mir411, Mir136, Mir431, Af357426, Af357425, Af357428, Af357341, Begain. | | | |

**Table B.**

| **Lineage** | **Imprinted genes**  **count** | **Lineage-specific imprinted genes** | **Plurigenes count** | **Lineage-specific plurigenes** | **Hematopietic genes count** | **Lineage-specific hematopietic genes** | **Lineage related phenotypes due to genes knock-out**  **(Not complete)** |
| --- | --- | --- | --- | --- | --- | --- | --- |
| **B-cell** | 27 | Ppp1r9a, Ndn, Slc22a3, Peg12, Sgce, Gatm, Cdkn1c, Gab1, Cmah, Asb4, Impact, Mkrn3, Tspan32, Phlda2, Cd81, Ddc, Mcts2, Tfpi2, Airn, Kcnq1ot1, Peg3, Sp2, Axl, Sfmbt2, Slc22a18, Nap1l4, Phf17 | 102 | Mpl, Smo, Ccnd1, Bmpr2, Relb, Gab1, Arid3b, Ctbp2, Rel, Tle2, Spp1, Tcf3, Mitf, Tcfeb, Lefty1, Klf2, Akt1, Creb1, Hcfc1, Mef2d, Smad1, Klf4, Ewsr1, Pik3cd, Tgfb1, Irs1, Pou2f1, Lef1, Psen1, Axin1, Rcn2, Dnmt3b, Pim3, Smarca4, Dhx9, Ehmt2, Mta2, Hras1, Kat5, Rif1, Stk40, Raf1, Sgk1, Myc, Zfx, Mbd3, Mapk1, Fgfr1, Hira, Smarca2, Zfp143, Carm1, Parp1, Acvr1b, Xpo4, Smarcad1, Ssrp1, P4ha1, Pias4, Satb2, Id1, Dffa, Paf1, Mycn, Ocln, Pbrm1, Rcor2, Wdr61, Fgf4, Wwp2, H3f3a, Smarcc1, Rbbp7, Grb2, Med12, Mtf2, Dnmt3a, Sumo1, Tcfe3, Ehmt1, Aes, Lyar, Smad4, Cdk2ap1, Il6st, Terf2, Chd4, Kdm4c, Ddb1, Smarca5, Phf17, Zfp57, Hdac1, Rela, Cdk2, Utf1, Hdac2, Grsf1, Ipo7, Smad2, Dnmt1, Acvr1 | 64 | Ccr7, Irf4, Meis1, Cd79a, Tmem176b, Fzd7, Tmem176a, Sox6, Hoxb3, Vnn1, Rbp1, Hoxa9, Ikzf3, Tgfbr3, Nbeal2, Prtn3, Dtx1, Pbx1, Dnaja3, Id2, Cd27, Polm, Pdgfrb, Dyrk3, Ccl5, Il7r, Fut7, Relb, Card11, Thsd1, Myo1e, Klf1, Il15, Rag2, Cxcr5, Slc40a1, Cebpa, Ahsp, Gfi1b, Gpr183, Flt3, Ccl3, Lta, Cd83, Lilrb3, Chd7, Il18r1, Angpt1, Tal1, Gata3, Kit, Spib, Ifnz, Tek, Gata2, H2-Ab1, Hdac5, Cd34, Pf4, Thoc5, Srf, Clec2i, Hlx, Trf | MP:0002144-abnormal B cell differentiation  MP:0004939-abnormal B cell morphology  MP:0004978-decreased B-1 B cell number  MP:0005093-decreased B cell proliferation  MP:0008024-absent lymph nodes  MP:0008209-decreased pre-B cell number  MP:0000702-enlarged lymph nodes  MP:0002023-B cell derived lymphoma  MP:0002401-abnormal lymphopoiesis  MP:0010763-abnormal hematopoietic stem cell physiology  MP:0008102-lymph node hyperplasia  MP:0004810-decreased hematopoietic stem cell number  MP:0010763-abnormal hematopoietic stem cell physiology  MP:0002459-abnormal B cell physiology  MP:0008174-decreased follicular B cell number  MP:0008470-abnormal spleen B cell follicle morphology  MP:0005154- increased B cell proliferation  MP:0004978- decreased B-1 B cell number |
| **Erythrocytes** | 4 | Sgce, Mkrn3, Kcnq1ot1, Sfmbt2 | 8 | Stat3, Rcn2, Mpl, Satb1, Mef2c, Acvr1b, Smad1, Hras1 | 11 | Tek, Add2, Fli1, Crip2, Rbp1, Gata2, Satb1, Cd27, Ahsp, Mef2c, Acvr1b | MP:0008973-decreased erythroid progenitor cell number  MP:0009395-increased nucleated erythrocyte cell number  MP:0002875-decreased erythrocyte cell number  MP:0000245-abnormal erythropoiesis  MP:0002447-abnormal erythrocyte morphology  MP:0003656-abnormal erythrocyte physiology  MP:0003657-abnormal erythrocyte osmotic lysis  MP:0002416-abnormal proerythroblast morphology  MP:0003131-increased erythrocyte cell number  MP:0003135-increased erythroid progenitor cell number |
| **Granulocytes** | 6 | Ppp1r9a, Sgce, Ndn, Peg12, Impact, Mkrn3 | 3 | Pml, Tert, Phc1 | 7 | Hoxa5, Gfi1b, Gata3, Rbp1, Angpt1, Meis1, Csf1r | MP:0000334-decreased granulocyte number  MP:0005072-abnormal hair follicle melanin granule morphology  MP:0000322-increased granulocyte number  MP:0002396-abnormal hematopoietic system morphology/development  MP:0002123-abnormal hematopoiesis  MP:0000715-decreased thymocyte number |
| **Monocytes** | 9 | Sgce, Peg12, Ndn, Ppp1r9a, Impact, Klrb1f, Mkrn3, Phlda2, Cdkn1c | 6 | Mpl, Tle2, Tert, Phc1, Rel, Smad7 | 19 | Cebpa, Csf1r, Egr1, Lgals1, Hoxa5, Gfi1b, Car2, Gata3, Rbp1, Angpt1, Gimap5, Tgfbr3, Pglyrp1, Meis1, Gata2, Sema4a, Nrarp, Tek, Junb | MP:0008112-abnormal monocyte differentiation  MP:0002445-abnormal mononuclear cell differentiation  MP:0000220-increased monocyte cell number  MP:0000223-decreased monocyte cell number  MP:0002123-abnormal hematopoiesis |
| **NK-cell** | 12 | Klrb1f, Ppp1r9a, Cdkn1c, Gab1, Ndn, Slc22a3, Sgce, Phlda2, Impact, Mkrn3, Cd81, Ampd3 | 16 | Mpl, Tcf7, Gab1, Relb, Lef1, Smo, Rif1, Mitf, Gatad2a, Klf2, Chd4, Rbl2, Sp1, Atrx, Axin1, Mycn | 45 | Lck, Rbp1, Fzd7, Ccr2, Cd28, Id2, Card11, Tcf7, Meis1, Txk, Ifng, Vnn1, Tbx21, Sox6, Eomes, Tesc, Cd3d, Ikzf3, Bcl11a, Prdm1, Relb, Lef1, Angpt1, Tiparp, Kit, Ccl3, Tal1, Gata2, Sema4a, Zap70, Dyrk3, Ccl5, Gab3, Lyl1, Hoxa9, Prtn3, Tgfbr3, Tek, Chd7, Dtx1, H2-Oa, Hdac9, Hlx, Polm, Rsad2 | MP:0008040-decreased NK T cell number  MP:0002339-abnormal lymph node morphology  MP:0002123-abnormal hematopoiesis  MP:0008047-absent uterine NK cells  MP:0008038-abnormal NK T cell number  MP:0008044-increased NK cell number  MP:0008045-decreased NK cell number  MP:0008046-absent NK cells |
| **T-cell** | 30 | Ndn, Ppp1r9a, Sgce, Peg12, Gab1, Asb4, Slc22a3, Mkrn3, LOC100505359, Cdkn1c, Phlda2, Cmah, Gatm, Impact, Igf2r, Tfpi2, Slc22a18, Nap1l4, Sfmbt2, Th, Peg3, Mcts2, Sp2, Dhcr7, Plagl1, Ddc, H13, Tspan32, Cd81, Xlr4c | 70 | Mpl, Tcf7, Ctbp2, Spp1, Gab1, Mef2c, Kdm6b, Zfp219, Tle2, Smad3, Lefty1, Ccnd1, Rcn2, Satb1, Smad1, Mitf, Lef1, Creb1, Pias4, Psen1, Mef2d, Fgfr1, Stk40, Klf2, Ocln, Socs1, Hras1, Ewsr1, Hdac1, Bmpr2, Mycn, Axin1, Ctcf, Aes, Grb2, Mbd3, Pim1, Ercc5, Hcfc1, Dnmt3b, Ehmt2, Pik3cd, Paf1, Mta2, Dhx9, Terf2, Ddb1, Med12, Gadd45gip1, Pim3, Smurf1, Tgfb1, Arid3b, Cdk2, Hira, Id1, Prkaca, Foxd3, Notch1, Hif1a, Il6st, Leo1, Tcf3, Smad2, Kat5, Acvr1b, Trp53, Atf2, Kdm6a, Dnmt3l | 53 | Lck, Fzd7, Rbp1, Tcf7, Gata2, Dtx1, Prtn3, Tek, Tnfsf11, Il15, Meis1, Zap70, Bcl11b, Cd3e, Kit, Srf, Cd3d, Vnn1, Car2, Tal1, Dyrk3, Tirap, Lyl1, Pf4, Tesc, Sema4a, Anxa1, Hoxa9, Mef2c, Angpt1, Il7r, Gfi1b, Themis, Lta, Hdac9, Gata3, Itk, Ctla4, Tnf, Cd34, Hhex, Hlx, Gpr183, Ccr7, Cd4, Tcra, Nkap, Thoc5, Il2ra, Trf, Il4, Tbx21, Eomes | MP:0002145-abnormal T cell differentiation  MP:0005018-decreased T cell number  MP:0008075-decreased CD4-positive T cell number  MP:0008079-decreased CD8-positive T cell number  MP:0008083-decreased single-positive T cell number  MP:0002123-abnormal hematopoiesis  MP:0008051-abnormal memory T cell physiology  MP:0002024-T cell derived lymphoma  MP:0008070-absent T cells |
| **Shared by all Lymphoid lineages**: Ppp1r9a, Ndn, Slc22a3, Sgce, Cdkn1c, Gab1, Impact, Mkrn3, Phlda2, Cd81, Meis1, Fzd7, Vnn1, Rbp1, Hoxa9, Prtn3, Dtx1, Dyrk3, Angpt1, Tal1, Kit, Tek, Gata2, Hlx, Mpl, Mitf, Klf2, Lef1, Axin1, Mycn | | | | | | | |
| **Shared by all Myeloid lineages**: Sgce, Mkrn3, Rbp1 | | | | | | | |
| **Exclusive in Lymphoid Lineages:** Slc22a3, Gatm, Gab1, Cmah, Asb4, Tspan32, Cd81, Ddc, Mcts2, Tfpi2, Airn, Peg3, Sp2, Axl, Slc22a18, Nap1l4, Phf17, Ampd3, LOC100505359, Igf2r, Th, Dhcr7, Plagl1, H13, Xlr4c, Ccr7, Irf4, Cd79a, Tmem176b, Fzd7, Tmem176a, Sox6, Hoxb3, Vnn1, Hoxa9, Ikzf3, Nbeal2, Prtn3, Dtx1, Pbx1, Dnaja3, Id2, Polm, Pdgfrb, Dyrk3, Ccl5, Il7r, Fut7, Relb, Card11, Thsd1, Myo1e, Klf1, Il15, Rag2, Cxcr5, Slc40a1, Gpr183, Flt3, Ccl3, Lta, Cd83, Lilrb3, Chd7, Il18r1, Tal1, Kit, Spib, Ifnz, H2-Ab1, Hdac5, Cd34, Pf4, Thoc5, Srf, Clec2i, Hlx, Trf, Lck, Ccr2, Cd28, Tcf7, Txk, Ifng, Tbx21, Eomes, Tesc, Cd3d, Bcl11a, Prdm1, Lef1, Tiparp, Zap70, Gab3, Lyl1, H2-Oa, Hdac9, Rsad2, Tnfsf11, Bcl11b, Cd3e, Tirap, Anxa1, Themis, Itk, Ctla4, Tnf, Hhex, Cd4, Tcra, Nkap, Il2ra, Il4, Smo, Ccnd1, Bmpr2, Arid3b, Ctbp2, Spp1, Tcf3, Mitf, Tcfeb, Lefty1, Klf2, Akt1, Creb1, Hcfc1, Mef2d, Klf4, Ewsr1, Pik3cd, Tgfb1, Irs1, Pou2f1, Psen1, Axin1, Dnmt3b, Pim3, Smarca4, Dhx9, Ehmt2, Mta2, Kat5, Rif1, Stk40, Raf1, Sgk1, Myc, Zfx, Mbd3, Mapk1, Fgfr1, Hira, Smarca2, Zfp143, Carm1, Parp1, Xpo4, Smarcad1, Ssrp1, P4ha1, Pias4, Satb2, Id1, Dffa, Paf1, Mycn, Ocln, Pbrm1, Rcor2, Wdr61, Fgf4, Wwp2, H3f3a, Smarcc1, Rbbp7, Grb2, Med12, Mtf2, Dnmt3a, Sumo1, Tcfe3, Ehmt1, Aes, Lyar, Smad4, Cdk2ap1, Il6st, Terf2, Chd4, Kdm4c, Ddb1, Smarca5, Zfp57, Hdac1, Rela, Cdk2, Utf1, Hdac2, Grsf1, Ipo7, Smad2, Dnmt1, Acvr1, Gatad2a, Rbl2, Sp1, Atrx, Kdm6b, Zfp219, Smad3, Socs1, Ctcf, Pim1, Ercc5, Gadd45gip1, Smurf1, Prkaca, Foxd3, Notch1, Hif1a, Leo1, Trp53, Atf2, Kdm6a, Dnmt3l | | | | | | | |
| **Exclusive in Myeloid Lineages:** Add2, Fli1, Crip2, Hoxa5, Csf1r, Egr1, Lgals1, Gimap5, Pglyrp1, Nrarp, Junb, Stat3, Pml, Tert, Phc1, Smad7 | | | | | | | |

**Table C.**

| **GOID** | **TERM** | **Total no of annotated genes in this term** | **Overlap with pluripotency genes** | **P-Value** | **ANNOTATED_GENES** |
| --- | --- | --- | --- | --- | --- |
| GO:0048534 | Hematopoietic or lymphoid organ development | 615 | 44 | 2.56E-20 | Mef2c, Satb1, Sp3, Cdc73, Psen1, Hand2, Relb, Tpo, Ctr9, Acvr1b, Tcfe3, Klf2, Tgfb1, Sp1, Hif1a, Notch1, Hells, Shh, Cdkn2a, Trp53, Tcf7, Apc, Nfkb1, Myc, Klf4, Leo1, Smarca4, Wnt5a, Tgfbr1, Mitf, Ctnnb1, Kdm1a, Jarid2, Lef1, Rbpj, Lrp5, Bmp4, Wdr61, Lif, Nme2, Pml, Paf1, Ogt, Wnt3a |
| GO:0030097 | Hemopoiesis | 574 | 39 | 7.09E-17 | Mef2c, Satb1, Sp3, Cdc73, Psen1, Relb, Tpo, Ctr9, Acvr1b, Tcfe3, Klf2, Tgfb1, Sp1, Hif1a, Notch1, Hells, Shh, Cdkn2a, Trp53, Tcf7, Apc, Myc, Klf4, Leo1, Smarca4, Wnt5a, Mitf, Ctnnb1, Kdm1a, Lef1, Rbpj, Bmp4, Wdr61, Nme2, Lif, Pml, Paf1, Ogt, Wnt3a |
| GO:0030099 | Myeloid cell differentiation | 275 | 27 | 6.83E-15 | Mef2c, Myc, Sp3, Cdc73, Psen1, Relb, Smarca4, Leo1, Ctr9, Acvr1b, Tcfe3, Ctnnb1, Mitf, Kdm1a, Lef1, Klf2, Tgfb1, Sp1, Hif1a, Bmp4, Wdr61, Lif, Nme2, Pml, Paf1, Ogt, Apc |
| GO:0045637 | Regulation of myeloid cell differentiation | 146 | 18 | 6.90E-11 | Mef2c, Myc, Cdc73, Leo1, Acvr1b, Ctr9, Tcfe3, Mitf, Ctnnb1, Kdm1a, Lef1, Hif1a, Wdr61, Nme2, Lif, Paf1, Ogt, Apc |
| GO:0002573 | Myeloid leukocyte differentiation | 144 | 16 | 1.01E-08 | Mef2c, Myc, Sp3, Psen1, Relb, Mitf, Ctnnb1, Tcfe3, Kdm1a, Lef1, Tgfb1, Bmp4, Nme2, Lif, Ogt, Apc |
| GO:0002521 | Leukocyte differentiation | 369 | 23 | 3.65E-08 | Mef2c, Myc, Satb1, Sp3, Psen1, Relb, Tcfe3, Mitf, Ctnnb1, Kdm1a, Lef1, Rbpj, Tgfb1, Bmp4, Lif, Nme2, Ogt, Hells, Shh, Cdkn2a, Trp53, Tcf7, Apc |
| GO:0046649 | Lymphocyte activation | 469 | 23 | 4.12E-06 | Cdkn1a, Mef2c, Satb1, Sp3, Psen1, Relb, Ctnnb1, Smad3, Lef1, Rbpj, Tgfb1, Bmp4, Hells, Shh, Pik3cd, Cdkn2a, Wnt3a, Trp53, Icam1, Il6st, Tcf7, Casp3, Apc |
| GO:0045321 | Leukocyte activation | 559 | 24 | 2.41E-05 | Cdkn1a, Mef2c, Satb1, Sp3, Psen1, Relb, Wnt5a, Ctnnb1, Smad3, Lef1, Rbpj, Tgfb1, Bmp4, Hells, Shh, Pik3cd, Cdkn2a, Wnt3a, Trp53, Icam1, Il6st, Tcf7, Casp3, Apc |
| GO:0071887 | Leukocyte apoptotic process | 65 | 9 | 8.52E-05 | Mef2c, Pten, Akt1, Myc, Bmp4, Cd44, Cdkn2a, Wnt5a, Trp53 |
| GO:0042110 | T-cell activation | 315 | 17 | 1.57E-04 | Satb1, Sp3, Psen1, Relb, Ctnnb1, Smad3, Lef1, Tgfb1, Bmp4, Cdkn2a, Shh, Trp53, Icam1, Il6st, Tcf7, Casp3, Apc |
| GO:0048538 | Thymus development | 37 | 7 | 3.31E-04 | Hand2, Jarid2, Shh, Tgfbr1, Apc, Psen1, Ctnnb1 |
| GO:0046651 | Lymphocyte proliferation | 197 | 13 | 6.16E-04 | Cdkn1a, Lef1, Mef2c, Satb1, Tgfb1, Bmp4, Shh, Cdkn2a, Wnt3a, Il6st, Trp53, Casp3, Ctnnb1 |
| GO:0070661 | Leukocyte proliferation | 206 | 13 | 1.03E-03 | Cdkn1a, Lef1, Mef2c, Satb1, Tgfb1, Bmp4, Shh, Cdkn2a, Wnt3a, Il6st, Trp53, Casp3, Ctnnb1 |
| GO:0045638 | Negative regulation of myeloid cell differentiation | 64 | 8 | 1.15E-03 | Paf1, Leo1, Myc, Ctr9, Wdr61, Cdc73, Ctnnb1, Nme2 |
| GO:0002761 | Regulation of myeloid leukocyte differentiation | 88 | 9 | 1.23E-03 | Lef1, Myc, Lif, Nme2, Ogt, Mitf, Ctnnb1, Tcfe3, Apc |
| GO:0030217 | T-cell differentiation | 177 | 12 | 1.36E-03 | Lef1, Satb1, Tgfb1, Sp3, Bmp4, Relb, Shh, Cdkn2a, Trp53, Tcf7, Apc, Ctnnb1 |
| GO:0030098 | Lymphocyte differentiation | 250 | 14 | 1.55E-03 | Lef1, Rbpj, Satb1, Tgfb1, Sp3, Bmp4, Relb, Hells, Shh, Cdkn2a, Trp53, Tcf7, Apc, Ctnnb1 |
| GO:0070227 | Lymphocyte apoptotic process | 52 | 7 | 3.75E-03 | Pten, Cd44, Akt1, Myc, Wnt5a, Trp53, Bmp4 |
| GO:0002260 | Lymphocyte homeostasis | 55 | 7 | 5.54E-03 | Mef2c, Cd44, Akt1, Pik3cd, Tgfb1, Casp3, Hif1a |
| GO:0070228 | Regulation of lymphocyte apoptotic process | 36 | 6 | 6.06E-03 | Pten, Cd44, Myc, Wnt5a, Trp53, Bmp4 |
| GO:0034101 | Erythrocyte homeostasis | 110 | 9 | 8.17E-03 | Kdm1a, Klf2, Axin1, Sp1, Sp3, Bmp4, Hif1a, Smarca4, Acvr1b |
